# Restoring Impaired Fertility Through Diet: Observations of Switching From High-Fat Diet During Puberty to Normal Diet in Adulthood Among Obese Male Mice

**DOI:** 10.3389/fendo.2022.839034

**Published:** 2022-04-19

**Authors:** Xiangyu Qi, Meijie Zhang, Mingqi Sun, Dandan Luo, Qingbo Guan, Chunxiao Yu

**Affiliations:** ^1^ Department of Endocrinology, Shandong Provincial Hospital, Cheeloo College of Medicine, Shandong University, Jinan, China; ^2^ Department of Endocrinology, Shandong Provincial Hospital Affiliated to Shandong First Medical University, Jinan, China; ^3^ Shandong Clinical Research Center of Diabetes and Metabolic Diseases, Shandong Provincial Hospital, Jinan, China; ^4^ Shandong Laboratory of Endocrinology and Lipid Metabolism, Shandong Provincial Hospital, Jinan, China; ^5^ Shandong Prevention and Control Engineering Laboratory of Endocrine and Metabolic Diseases, Shandong Provincial Hospital, Jinan, China; ^6^ Jing’an District Center Hospital, Fudan University, Shanghai, China; ^7^ General Practice, Jinan Forth People’s Hospital, Jinan, China

**Keywords:** obesity, high fat diet, restoring to low fat diet, puberty, testis, fertility

## Abstract

**Background:**

Obesity is associated with a decrease in testicular function, yet the effects and mechanisms relative to different stages of sexual development remain unclear. The aim of this study is to determine whether high-fat diet-induced obesity impairs male fertility during puberty and in adulthood, and to ascertain its underlying mechanisms. This study aims to further reveal whether restoring to a normal diet can improve impaired fertility.

**Methods:**

Male mice were divided into 6 groups: the group N and H exposed to a normal diet or high-fat diet during puberty. The group NN or NH were further maintained a normal diet or exposed to high-fat diet in adulthood, the group HH or HN were further maintained high-fat diet or switched to normal diet in adulthood. Metabolic parameters, fertility parameters, testicular function parameters, TUNEL staining and testicular function-related proteins were evaluated, respectively.

**Results:**

The fertility of the mice in the high-fat diet group was impaired, which validated by declines in pregnancy rates and litter weight loss. Further analysis demonstrated the increased level of oxidative stress, the increased number of spermatogenic cell apoptosis and decreased number of sperm and decreased acrosome integrity. The expression of steroidogenic acute regulatory (StAR) and spermatogenesis related proteins (WT-1) decreased. Fertility among the HN group recovered, accompanied by the recovery of metabolism, fertility and testicular function parameters, StAR and WT-1 expression.

**Conclusions:**

The findings suggest that high-fat diet-induced obesity impairs male fertility during puberty and in adulthood. The loss of acrosome integrity, the increase of oxidative stress, the increase of cells apoptosis and the down-regulation of StAR and WT-1 may be the underlying mechanisms. Switching from high-fat diets during puberty to normal diets in adulthood can improve male fertility.

## Introduction

Over the past few decades, obesity has become a predominant global public health problem. Estimates of obesity in adults (defined as having a body mass index (BMI) ≥ 25 kg/m^2^) remain high, at 38.9% globally (1). Concurrently, obesity in childhood and puberty have also become an increasingly serious issue. In many Western countries, 12% - 18.5% of youth aged 2 - 19 years are classified as obese (2, 3). Clinical studies have shown that adult obesity precipitates a marked decline in sperm quality, which may be related to impaired sperm DNA and reduced embryo implantation rates (4–7). It is worth noting that a recent prospective cohort study showed that the offspring quantity among obese male adolescents reduced by 32% to 38% compared with normal-weight adolescents when they reached adulthood 21 years later (8). However, this is contradicted by another prospective longitudinal study spanning over two decades, which reported that there was no significant association between the prepubertal BMI and adult semen characteristics, even if serum reproductive hormone levels altered during adulthood (9). Given these conflicting findings, the effect of obesity during puberty on fertility remains unknown and controversial.

As a vital organ of the reproductive system, the testes are composed of Leydig cells (LCs) and seminiferous tubules. The latter contains spermatogenic cells and Sertoli cells (SCs). Through childhood and leading up to puberty, is an important stage in testicular development. Spermatogenesis starts at puberty, which is a finely regulated process of germ cell multiplication and differentiation, leading to the production of spermatozoa in the seminiferous tubules (10, 11). As a continuous process, spermatogenesis is maintained by testosterone secreted in LCs, protected and nutritionally supported by SCs (12, 13). During puberty, some remarkable developmental changes occur in morphology and physiology leading to sexual maturity, then entering into adulthood. It worth to noting that previous studies showed that obesity induced by high-fat diet impaired spermatogenesis *via* the dysfunction of LCs and decreased viability of SCs in adult male mice. Hence, given that the development and maturity of pubertal testes are essential to normal reproductive function, it is of importance to study the influence of pubertal obesity on testicular structure and function.

Obesity can be reversed by lifestyle changes, such as reducing calorie intake. A number of studies have shown that limiting caloric intake can significantly reduce the weight and improve metabolic health among obese men (14, 15). Recently, further studies have confirmed that reduced caloric intake in men classified as obese can not only help to reduce obesity, but also improve reproductive function; weight loss caused by reduced caloric intake has been associated with the increase in total sperm count and semen volume in men (16). Animal studies have shown that adverse sperm physiology observed in high-fat diet-fed mice, such as decreased sperm motility and morphology, can be reversed after restoring normal diets (17). However, whether any return to normal diet has the potential to alleviate puberty-related obesity and improve reproductive function remains unclear, and further research is needed.

In this study, in order to study the effect of obesity on male reproductive function during sexual developmental stages, male mice were fed with a high-fat diet during puberty and in adulthood (boundary: 8 weeks) to establish a diet-induced obesity mouse model. The fertility, sperm quality and hormone levels, lipid and glucose metabolism, histopathological changes and testis-related proteins expression were further assessed. In addition, the extent to which restoring a normal diet alleviates the damage to fertility caused by obesity in puberty is further evaluated.

## Materials and Methods

### Animals and Diets

Three-week-old C57BL/6 male mice and eight-week-old female mice were purchased from Vital River Corporation (Beijing, China) and housed in constant temperature-controlled rooms with a 12-hour light/dark cycle. This study was approved by the Animal Ethics Committee of Shandong Provincial Hospital.

As shown in **Supplemental Figure 1**, male mice were randomly divided into two groups: the normal diet-group (N, n = 24) was fed a normal diet including 10 kcal% fat (D12450B, Research Diets, Inc, USA) during puberty, the high-fat-diet group (H, n = 24) was fed with high-fat diet containing 60 kcal% fat during puberty (D12492, Research Diets, Inc, USA). At the end of the 8 weeks, 8 mice in each group were sacrificed. The remaining mice in group N were further subdivided into two subgroups. The first subgroup maintained the normal diet (NN, n = 8) in adulthood, the second subgroup was fed with high-fat diet (NH, n = 8) in adulthood. Similarly, the remaining male mice of group H were divided into two subgroups. One subgroup maintained high-fat diet (HH, n = 8) in adulthood, the other subgroup was fed with normal diet (HN, n = 8) in adulthood. The body weights of the mice were monitored weekly during the feeding period. All mice were sacrificed at the end of 16 weeks.

After being fasted for 8 h, all mice were anesthetized with pentobarbital sodium and sacrificed. The blood was collected from the retroorbital vein of anesthetized mice; after standing for 40 min, blood was centrifuged at 5,000 rpm for 20 min, and the serum supernatants were stored at −80°C until measurement. Epididymal tails were collected immediately, testes and epididymal fat were separated and weighed. Testis coefficient was estimated by the testes weight/bodyweight. Then the testes were rapidly preserved in liquid nitrogen for oil red staining and protein analysis, or in Modified Davidson’s fluid (mDF, containing 30% of a 37-40% solution of formaldehyde, 15% ethanol, 5% glacial acetic acid and 50% distilled water) for morphological analysis, including haematoxylin and eosin (H&E) and TUNEL staining.

### Reproductive Ability Assay

To evaluate the reproductive ability of male mice with different feeding patterns, each male mouse was mated with two 8-week-old female mice during 5 pm to 8 am for five consecutive days at the age of 8 or 16 weeks. All mice were fed with normal diet during mating period, and vaginal plugs of female mice were tested every morning. Males and females were separated from 8 am to 5 pm every day, and high-fat diet was given to the males in H, NH or HH group. After the 5 days mating period, the males in every group were fed normal diet or high-fat diet respectively for two days before being euthanized. The female mice with vaginal plugs were moved to another cage and observed until the pups were born. The number of pregnant females, the number and weight of the offspring were recorded.

### Measurement of Lipid Profile and Sex Hormone

The serum levels of glucose (GLU) and lipid profiles containing triglyceride (TG), total cholesterol (TC), low-density lipoprotein cholesterol (LDL-c) and high-density lipoprotein cholesterol (HDL-c) were measured by the biochemical analyser (Mindray Bio-medical Electronics co. LTD). The levels of testosterone in serum were detected with Enzyme-Linked Immunosorbent Assay (ELISA) Kit (CUSBIO, Wuhan, China) according to the kit manufacturer’s protocol. The detection limit of testosterone ELISA is 0.1-20 ng/ml, the inter-/intra-coefficient of variability is less than 15% (CV% < 15%). All the serum samples were tested in duplicate. The MDA content of testis was used the kits (Beyotime Biotechnology, China).

### Sperms Parameters Detection

The epididymal tails of the mice were placed in M199 (Hyclone, USA) with 0.5% BSA. In the preheated petri dishes, the epididymal tails were cut 5 times with scissors, and were immediately put into 37°C 5% CO_2_ incubator. After incubation for 3 min, the sperm suspension was blew and mixed gently. 5 μl sperm suspension was added into 100 μl M199 (the mixture was diluted according to the proportion of 1:20). Then 10 μl diluted sperms suspension was dropped on the sperm counting plate. The sperms concentration, movement of sperm motility and proportion of motile sperms were measured by CASA IVOS II (Hamilton-Thorne Bioscience, Beverly, MA, USA), and their activities were calculated. Each sample was counted at least 3 times.

### Acrosome Staining

To determine acrosome reaction and integrity of sperm, mice sperms were added to PNA-FITC and Hochest dye reagent (BYX1403C, Byxbio, China), then the mixture was placed in a wet box and stained with dye reagent for 1.5 h, washed with distilled water for 3 min, added the quenching agent. Images were captured by fluorescence microscopy imaging system (Axio Imager A2, Zeiss, Germany) and the percentage of sperms with acrosome loss was calculated. The green dots represented intact sperm acrosome, the blue dots represented sperm, (1-the number of green points/blue points) × 100% was the percentage of sperms with acrosome loss.

### Haematoxylin and Eosin (H&E) Staining

To verify the histopathological changes of the testis, testicular tissue samples were dehydrated in a graded series of ethanol solutions and embedded in paraffin. The paraffin blocks were sectioned to thickness of 5 μm using a microtome (Leica, Germany). H&E stained slides were scaLLed with the light microscopy imaging system (Imager A2, Zeiss, Germany).

### Oil Red Staining

To detect the accumulation of testicular lipid, 5 μm thickness frozen testes slices were fixed in 95% alcohol for 10 s, soaked in distilled water for 10 s and rinsed with running water for 2 min, incubated in the oil red dye for 15 min in the dark, counterstained with haematoxylin for 30 s. Representative photomicrographs were captured with the light microscopy imaging system.

### Protein Extraction and Immunoblot Analysis

Frozen testicular samples were homogenized in RIPA buffer with protease inhibitors and PMSF (Shenergy Biocolor Bioscience & Technology Company, Shanghai, China), then centrifuged at 12, 000 g for 15 min. The protein concentration was quantified using a BCA assay kit (Pierce Biotechnology, Inc., IL, USA). A total of 60 μg protein samples were subjected to 10% SDS-PAGE, electro transferred to PVDF membrane (Millipore, Billerica, MA, USA). The membranes were blocked with 5% non-fat dry milk in Tris-buffered saline (TBS) for 1 h, and then incubated with specific primary antibodies StAR (1:1000, 8449S, CST), WT-1 (1:1000, 83535S, CST) and DAZL (1:1000, 12633-1-AP, Proteintech) overnight at 4°C. GAPDH (1:2500, Proteintech, 60004-1-Ig) was used as standard control. The appropriate secondary antibodies (diluted according to the proportion of 1:5000) that conjugated to horseradish peroxidase (HRP) (Amersham, Little Chalfont Bcuks, UK) were used. The protein expression levels were detected and quantified with Fluor Chem Q SA software (ProteinSimple, San Jose, CA).

### Statistical Analysis

Statistical analyses were performed using GraphPad Prism 8. Differences between two groups were compared using an unpaired Student’s t-test. Two-way ANOVAs was used to compare the means of multiple groups. All data were expressed as Means ± SEM, and the differences were considered as statistically significant if p < 0.05.

## Results

### High-Fat Diet During Sexual Development Increases Body Weight and Epididymal Adipose Deposition

**Figure 1:**
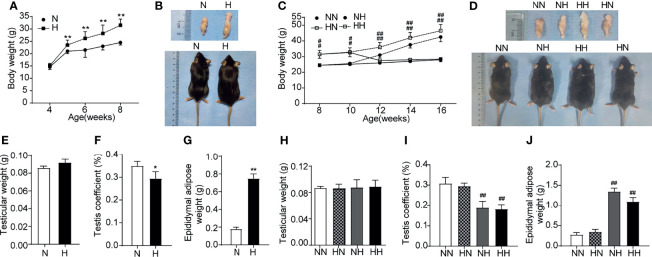
General conditions induced by high-fat diet during sexual development **(A)** Comparison of time-dependent changes in body weight between normal diet (N) and high-fat diet (H) group of 8-week age mice (n = 8 for each group). **(B)** Representative gross inspection of normal diet of (N) and H group at the 8th week. **(C)**. Comparison of time-dependent changes in body weight among NN group, HN group, NH group and HH group at 16th week (n = 8 for each group). **(D)** Representative gross inspection of NN group, HN group, NH group and HH group at the 16th week. **(E)** testicular weight and **(F)** testicular coefficient and **(G)** epididymal adipose in normal diet group (N) and high-fat diet group **(H)** of 8th week of feeding (n = 8 for each group). The comparison of **(H)** testicular weight and **(I)** testicular coefficient and **(J)** epididymal adipose among NN group, HN group, NH group and HH group at 16th week (n = 3 for each group). Results are presented as mean ± SEM. *p < 0.05, **p < 0.01 vs the N group. #p < 0.05, ##p < 0.01 vs the NN group.

To analyse the effects of a high-fat diet on general characteristics of mice, the body weights of mice were examined on a weekly basis over a total period of 8 or 16 weeks. **Figure 1A** illustrates how, during puberty, the weight of mice in H group reached their maximum weight by 8 weeks of age compared with the N group (*P* < 0.01). The representative photographs of mice are shown in **Figure 1B**. By 16 weeks, the weight of both NH and HH group were higher than that of NN group, the difference being statistically significant (*P* < 0.01). As expected, the average weight of the HH group was the highest due to the continuous high-fat diet from puberty to adulthood (see **Figures 1C, D**).

To evaluate the influence of this high-fat diet on the general characteristics of the testis, testicular weight, testis coefficient and epididymal adipose were recorded for all groups after 8 weeks and 16 weeks. The representative photographs of testis/epididymis are shown in **Figure 1B**. Although no clear difference was identified in the testicular weight among all groups (see **Figures 1E, H**), the testis coefficients in the H, NH and HH groups were significantly lower than those in the N or NN group (*P* < 0.05, **Figures 1F, I**). The epididymal adipose in H, NH and HH groups were significantly heavier than those in the N or NN groups (*P* < 0.01, see **Figures 1G, J**). These results indicate that obese mice models induced by high-fat diets during puberty and in adulthood were successfully established. Of note, at 16 weeks, the body weight and epididymal adipose of members in the HN group reduced to the normal level with NN group after restored to the normal diet from 8 weeks (**Figures 1C, D, J**).

### High-Fat Diet During Sexual Development Induces Abnormalities in Serum Glucose and Lipid Metabolism

**Figure 2:**
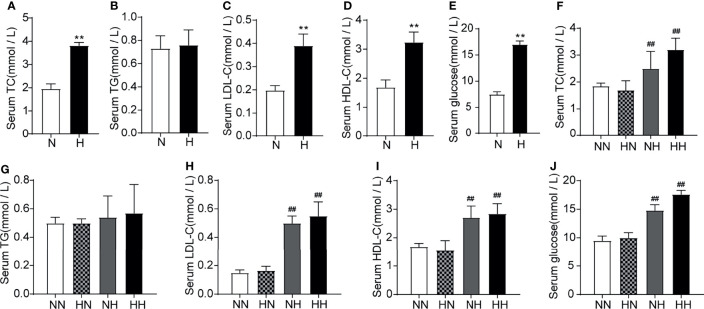
Lipid profile induced by high-fat diet during sexual development the level of serum TC, TG, LDL-C, HDL-C and serum glucose between normal diet (N) and high-fat diet (H) group of 8-week old mice (n = 8 for each group). **(A–E)**. The level of serum TC, TG, LDL-C, HDL-C and serum glucose of 16-week old mice among NN group, HN group, NH group and HH group (n = 8 for each group). **(F–J)** Results are presented as mean ± SEM. **p < 0.01 vs the N group. ##p < 0.01 vs the NN group.

Obesity is often accompanied by dyslipidaemia or hyperglycaemia (18, 19). To assess the effects of high-fat diets on glucose and lipid metabolism at different stages of sexual development, the circulating lipid profiles and glucose levels of all group were tested. As shown in **Figures 2A–E**, at 8 weeks, the serum TC, HDL-c, LDL-c and GLU levels of the high-fat diet fed mice increased significantly relative to normal diet fed mice (*P* < 0.01, respectively). Similar results were found at 16 weeks (**Figures 2F–J**). It is worth noting that serum glucose and lipid metabolism parameters of the HN group decreased significantly relative to HH group among which the serum TG, HDL-c, LDL-c and GLU fell back to normal levels with NN group. These findings demonstrate that the glucose and lipid metabolism are substantially disrupted as a result of a high-fat diet. Subsequently, restoring the mice to a normal diet in adulthood significantly alleviated the disorder of glucose and lipid metabolism during puberty.

### Restoring to Normal Diet in Adulthood Improves the Impaired Sperm Quality and Fertility Induced by High-Fat Diet During Puberty

**Figure 3:**
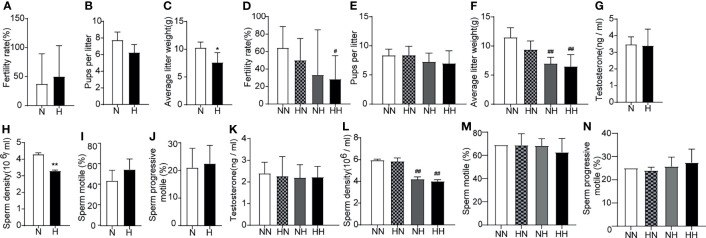
Reproduction function parameters and acrosome reaction and integrity induced by high-fat diet during sexual development Fertility **(A)**, pups per litter **(B)** and average litter weight **(C)** were observed, testosterone levels **(G)** (n = 8 for each group), sperm parameters **(H–J)** (n = 4 for each group) were assayed at the 8th week of feeding. Fertility **(D)**, pups per litter **(E)** and average litter weight **(F)** were observed, testosterone levels **(K)** and sperm parameters **(L–N)** were assayed at the 16th week of feeding (n = 8 for each group). *p < 0.05, **p <0.01 vs the N group. #p < 0.05, ##p < 0.01 vs the NN.

First, to assess the effect of high-fat diets on the fertility during sexual development, the reproductive abilities of the male mice were assessed *via* cohabitation with female mice. At the 8^th^ week, although no difference in the fertility rate or the number of pups per litter between N and H groups was ascertained (**Figures 3A, B**), the average litter weight of H group was significantly lower than that of N group (*P* < 0.05; **Figure 3C**). This data suggests that a high-fat diet during puberty affected the weight of offspring. By the 16^th^ week, the fertility rate of the HH group was lower than of the NN group (**Figure 3D**). Although there was no difference in the number of pups, the average litter weight of the NH and HH groups were significantly lower than that of the NN group (*P* < 0.01, respectively; **Figures 3E, F**). In order to further analyse the potential causes for this diminished fertility, serum testosterone and sperm parameters were examined. Although no significant difference was manifest in serum testosterone levels and most sperm parameters among mice at both 8 weeks and 16 weeks (**Figures 3G, I–K, M, N**), the sperm density in H group was lower than that of N group (*P* < 0.01, **Figure 3H**) at the 8^th^ week. At the 16^th^ week, the decline of the NH and HH group worsened to more than that of the NN group, (*P* < 0.01, respectively; **Figure 3L**). These results suggest that a high-fat diet in both puberty and adulthood impaired reproductive ability.

**Figure 4:**
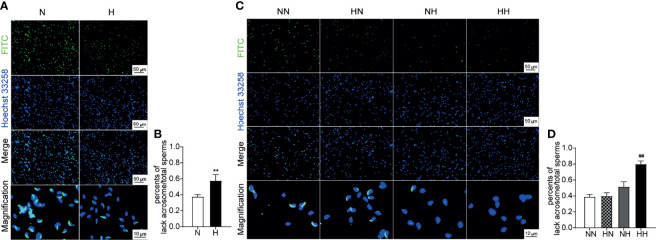
Acrosome reaction and integrity induced by high-fat diet during sexual development Testicular tissue sperm PNA—FITC staining of 8-week and 16-week old mice **(A, C)**. The percentage of lack of acrosome were calculated **(B, D)** (n = 3 for each group). Results are presented as mean ± SEM. **p < 0.01 vs the N group. ^##^p < 0.01 vs the NN group.

The sperm quality was further tested by the PNA-FITC staining sperm acrosome reaction experiment (20). The integrity of sperm acrosome is an important prerequisite for fertilization; sperm acrosome defects or weak acrosome reactions have the potential to result in fertilization disorders (21). By week 8, the percentage of sperm with acrosome loss of H group was significantly higher than that of N group (*P* < 0.01; **Figures 4A, B**). Similarly, at the 16^th^ week, the percentage of sperm with acrosome loss of HH group was significantly higher than that of NN group (*P* < 0.01; **Figures 4C, D**). These results indicate that a high-fat diet significantly impaired the sperm structural integrity.

Most notably, after being restored to the normal diet, the average litter quantity and weight, the sperm density and the acrosome integrity of the HN group returned to normal levels. These results suggested that reverting to the normal diet in adulthood can significantly alleviate fertility disorders caused by the high-fat diet during puberty.

### Reverting Back to the Normal Diet in Adulthood Relieves Testicular Lipid Ectopic Deposition and Morphological Abnormalities Induced by the High-Fat Diet During Puberty

**Figure 5:**
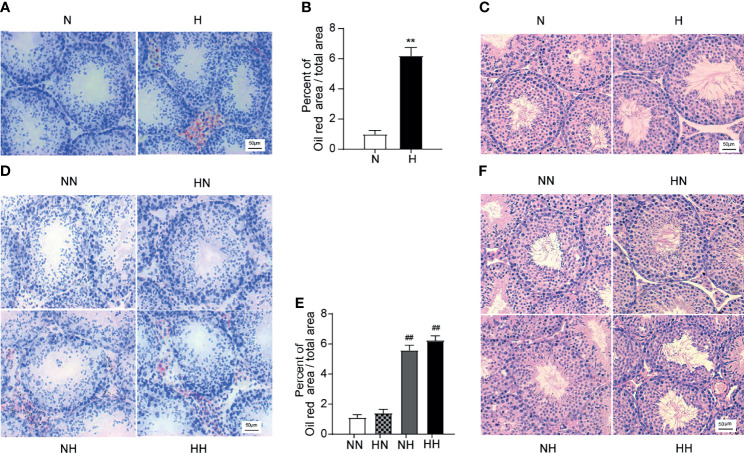
Testicular lipid deposition and morphological induced by high-fat diet during sexual development **(A)** Oil red O stained testicular sections at the 8th -week age of mice, and lipid abundance in the testis was analysed **(B)** (n = 3 for each group). **(C)** Oil red O stained testicular sections at the 16-week age of mice, and lipid abundance in the testis was analysed among NN group, HN group, NH group, HH group **(D)** (n = 3 for each group). **(E)** Representative photomicrographs of H&E staining of testicular sections at 8th week were shown, H&E stained testicular sections at the 16th week of feeding were observed among NN group, HN group, NH group, HH group **(F)** (n = 3 for each group). All of the above micrographs were from at least three independent experiments, magnification were 200 times. Results are presented as mean ± SEM. **p < 0.01 vs the N group. ^##^p < 0.01 vs the NN group.

To determine the effect of the high-fat diet and subsequent reversion to the normal diet on lipid accumulation in the testis, the frozen sections were stained with the red oil O. By week 8, the testicular tissue of H group exhibited higher lipid values and abnormal lipid accumulation in Leydig cells and seminiferous tubules (*P* < 0.01; **Figures 5A, B**). Consistent with the above results, the lipid content in testicular tissues of the NH and HH groups was significantly higher than that of the NN group (*P* < 0.01, respectively; **Figures 5D, E**). In order to further detect the morphological changes of the testis, testicular paraffin sections were evaluated by H&E staining. As shown in **Figures 5C**, by the 8^th^ week, the seminiferous tubules were arranged loosely, the seminiferous epithelia was thin and the layer of spermatogenic cells (spermatogonia and spermatocytes) were damaged in testis of H group relative to N group. By week 16, regressive changes in the seminiferous tubules were further evident in the NH and HH groups (**Figure 5F**). The tubules were lined with a thin layer of fewer spermatogonia and spermatocytes, the sperm numbers in the lumen were particularly low and intraepithelial vacuoles were also observed in the tubules. These results indicate that lipid accumulation - at any stage - disrupts the general morphology of the testis and is likely to affect the function of the testis.

For the changes resulting from reverting back to the normal diet group, no significant difference between the HN group and the NN group is ascertained. Rather, the testicular lipid content and abnormal lipid accumulation in the HN group reduced to within normal levels after reverting to a normal diet in adulthood.

### Reverting to a Normal Diet in Adulthood Alleviates the Increased Level of Oxidative Stress and the Increased Number of Spermatogenic Cells Apoptosis Induced by High-Fat Diet During Puberty

**Figure 6:**
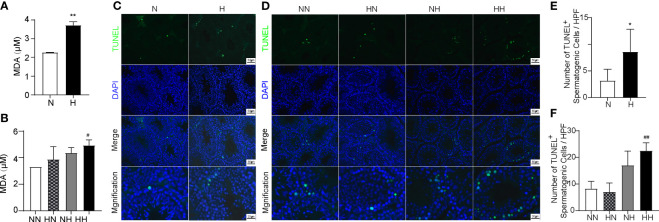
Oxidative levels and germ cell apoptosis induced by high-fat diet during sexual development. Testicular MDA content of 8-week and 16-week old mice **(A, B)** Testicular tissue TUNEL staining of 8-week and 16-week old mice **(C, D)**. The TUNEL+ cells/filed of spermatogenic cells were calculated **(E, F)** (n = 3 for each group). Arrows show TUNEL-positive spermatogonia, arrows head show TUNEL-positive primary spermatocyte. Results are presented as mean ± SEM. *p < 0.05, **p < 0.01 vs the N group. #p < 0.05, ##p < 0.01 vs the NN group.

Several studies have indicated that increased oxidative stress and excessive apoptosis of testicular tissue plays important roles in male spermatogenesis dysfunction; further analysis has corroborated this, by demonstrating that a high-fat diet accelerates the oxidative stress and apoptosis of testicular cells (22). In order to explore whether oxidative stress increased in high-fat diet feeding mice during sexual development, we measured the MDA content of testis using the kits. By week 8, the MDA of H group exhibited higher levels (P < 0.001; **Figure 6A**). By week 16, similar changes were identified in the HH group compared with the NN group (P<0.05, **Figure 6B**). Although there was no difference between HN and HH group, there was a decreasing trend in the MDA levels. In order to explore whether spermatogenic cells apoptosis high-fat induced fertility damage during sexual development, testes were stained with TUNEL. As illustrated in **Figure 6**, cell apoptosis is found to be primarily localised in the seminiferous epithelium, and the number of TUNEL-positive spermatogenic cells were counted. By weak 8, the number of apoptotic spermatogenic cells in the H group were more than that in the N group (*P* < 0.5; **Figures 6A, C**). By week 16, the number of apoptotic spermatogenic cells in the NH and HH group was more than that of the NN group (*P* < 0.01, respectively; **Figures 6B, D**). However, the number of apoptotic spermatogenic cells in the HN group did not change compared with the NN group. These results indicate that the high-fat diet both in puberty and adulthood induced the increased oxidative stress and extra apoptosis of spermatogenic cells, while restoring a normal diet in adulthood can significantly alleviate the abnormal oxidative stress and apoptosis exacerbated by the high-fat diet during puberty.

### Reverting to a Normal Diet in Adulthood Improves the Diminished Expression of Testosterone Synthesis and Spermatogenic-Related Enzymes Induced by a High-Fat Diet During Puberty

**Figure 7:**
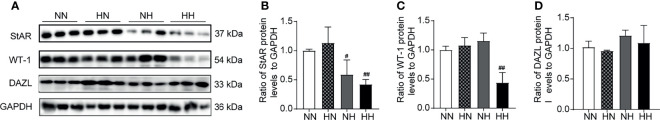
The expression of synthetic testosterone and spermatogenic enzymes induced by a high-fat diet during sexual development. **(A)** The bands of Western blotting analysis of StAR, WT-1, DAZL in mice testes. Testis lysates (40 µg/well) were loaded onto gel and reacted with primary and later secondary antibodies; GAPDH was set as loading control. The panel **(B–D)** is a densitometric histogram of protein bands and results were expressed as ratio of corresponding protein to GAPDH (n = 3 for each group). Results are presented as mean ± SEM. #p < 0.05, ##p < 0.01 vs the NN group. All of the above panels are representative of 3 independent experiments.

Spermatogenesis are complex processes, which maintained by testosterone secreted in Leydig cells (LCs), protected and nutritionally supported by Sertoli cells. StAR is the key enzyme for testosterone synthesis within the Leydig cells. WT-1 is a spermatogenesis-related protein and a specific marker of Sertoli cells. It plays an important role in gonadal development and sexual differentiation. DAZL, as the marker of spermatogenic cell, is essential for the proliferation and differentiation of germ cells (23, 24). As illustrated in **Figures 7A–D**, compared with the NN group, the expression of StAR significantly decreased in the NH and HH groups (*P* < 0.05, *P* < 0.01, respectively), the expression of WT-1 significantly decreased in the HH group (*P* < 0.01), while the expression of DAZL was similar in all groups. The expression levels of all proteins in the HN group were similar to those of the NN group. These results indicate that a continuous high-fat diet inhibits the expression of StAR and WT-1, while the simple high-fat diet during puberty also inhibited the expression of StAR. After reverting back to the normal diet in adulthood, abnormal protein expression returns to normal levels.

## Discussion

The number of individuals considered to be overweight or obese is growing rapidly, with the problem of obesity in children and adolescents become increasingly pertinent (25). An increasing number of studies have demonstrated that obesity is associated with male infertility, leading to empirically affirmed declines in testicular function in adult men (4, 26–28). However, studies focusing on the effects of obesity on the reproductive development and reproductive capacity of adolescents are controversial and presently lack in-depth investigation.

Puberty is a crucial period for the development of the male reproductive system. Testicular size doubles from early childhood to pre-puberty, after which the testicular volume reaches the growth peak during puberty and adults (29, 30). The rapid growth of the testicles during puberty is primarily facilitated by the expansion of germ cells and the increase in the seminiferous tubule diameter triggered by testosterone (31). During this process, the male reproductive system is more susceptible to external factors, such as hyperglycaemic or hyperlipidaemia, that risks altering reproductive function (32). Mice reach sexual maturity after 8 weeks of age, forming the boundary between puberty and adulthood (33). In this research., the effects of high-fat induced obesity on the fertility of mice different stages of sexual development, especially during puberty, were investigated.

Obesity is characterized by weight gain, often accompanied by the disorders of glycolipid metabolism (34, 35). In this study, the obesity model of mice was established by administering a high-fat diet. The high-fat diet mice at different stages all experienced weight gain, abnormal glucose and lipid metabolism. These proved the successful construction of a high-fat diet-induced obese mouse model. Apart from dyslipidemia of male mice, lipid accumulations were also deposited in the epididymal fat and testis. In the testis, a large amount of ectopic lipids accumulated in the testicular interstitium. Some lipids penetrated the basement membrane and deposited ectopically in the basement compartment of the seminiferous tubules. These results indicate that the testis and seminiferous tubules were in a lipotoxic microenvironment, which may lead to the impaired spermatogenesis.

The synthesis and secretion of testosterone occurs in the Leydig cells. Cholesterol enters the Leydig cells and is catalysed by the key enzymes (including StAR) to ultimately generate testosterone eventually (36–38). In this study, although there was no significant differences in serum testosterone among all groups, the protein expression of StAR in the NH and HH groups was significantly lower than that in the NN group. Our previous studies have confirmed that a high-fat diet would lead to significant decrease in testosterone levels in the testes (39). These suggested that a long-term high-fat diet (or a high-fat diet in adulthood) inhibits testosterone synthesis.

Sertoli cells provide nutrition and BTB (blood-testis-barrier) supports in spermatogenesis (40). Disrupting the integrity of BTB may result in reproductive dysfunction (13, 41). Our previous study has demonstrated that the integrity of BTB of high-fat diet-fed mice is disrupted (42). In this present study, compared to N group, these obese mice of H group exhibited epithelium of spermatogenic tubules which were thinner, the seminiferous epithelia disorganised, and cell adhesion between Sertoli cells and spermatogenic cells disrupted and loosely arranged. These damages were more pronounced in the NH and HH group. It’s worth noting that the protein expression levels of WT-1 in the HH group were significantly lower than those in NN group. These results indicate that the lipotoxicity induced by the high-fat diet risks impairing BTB and spermatogenesis. A continuous high-fat diet further inhibited WT-1 protein expression and ultimately led to reproductive dysfunction.

Studies have also shown that various factors such as heat, an unhealthy diet and pollutants can affect sperm quality. Our previous research has demonstrated that the sperm concentration decreased in obese male rats (43). A study from the Utah Population Database found that poor quality semen analysis parameters pointed towards an association with low birth weights in offspring (44). This may be caused by metabolic disorder in the offspring of sub-fertile men (45). This study found that high-fat diet mice, including the H, NH and HH groups, exhibited low sperm quality, which can be demonstrated by the decrease in sperm density and average litter weight. HH group exhibited even further declines in fertility. This indicates that high-fat diet manifests a certain degree of damage to sperm quality, but did not immediately cause male infertility. The long-term effects of high-fat diet certainly impaired male fertility. Sperm acrosome is an important structure for sperm fertilisation; its structural integrity plays a vital role in ensuring the normal function of sperm. Only sperm with an intact acrosome and capable of acrosome reaction can penetrate the zona pellucida (46). Previous studies have reported that male infertility is closely related to acrosomal membrane structure damage and to sperm ultrastructural abnormalities (47–49). Our study found that the acrosome integrity of high-fat diet mice during puberty was significantly lower than that of normal diet mice. The damages of sperm acrosome integrity among high-fat diet mice, especially in HH group. This may at least partly explain the decline in fertility in high-fat diet induced obese mice.

Oxidative stress is highly correlated with a wide variety of inflammatory and metabolic disease states, including obesity (42). MDA is an aldehyde generated in the process of lipid peroxidation caused by free radicals. MDA indicates cell membrane damage and reflects the severity of an oxygen radical attack on reactive cells and the levels of free radical metabolism *in vivo*. Increased MDA can trigger oxidative stress, causing cell damage and even death (50). In present study, to determine the effect of obesity on oxidative stress, the concentrations of MDA in testis homogenate were determined. In the present study, the content of MDA was found to have increased significantly in mice exposed to a high-fat diet, both in H and HH groups. This suggests that the increased level of oxidative might be involved in the fertility damage of high-fat diet mice.

Apoptosis plays an essential role in spermatogenesis. Physiological apoptosis can maintain the balance of spermatogenic cells to ensure appropriate sperm production during spermatogenesis (50–52). It has been demonstrated that the increased number of spermatogenic cell apoptosis is one of the most important drivers underpinning the production of low quantities of (or no) sperm, resulting in male infertility (53). In the present study, the apoptosis of spermatogenic cells was found to have increased significantly in mice exposed to a high-fat diet, both in HN and NH groups, whereas the apoptosis further increased in HH group. This suggests that the increased number of spermatogenic cell apoptosis might be involved in the fertility damage of high-fat diet mice. DAZL, as the marker of the spermatogenic cell, is essential for the proliferation and differentiation of germ cells. However, the protein expression level of DAZL in the high-fat diet mice at different periods was no different from that of the normal diet mice. This finding indicates that DAZL may not participate in the regulation of spermatogenic cells in high-fat diet mice.

Reverting to a normal diet is an effective way to alleviate obesity (54). However, it is unclear whether the fertility damage caused by obesity during puberty can be improved after returning to a normal diet in adulthood. In this study, after returning to a normal diet in adulthood, obesity in the HN group was significantly improved, accompanied by the significantly ameliorated dyslipidemia and testicular ectopic lipid. This suggests that reverting to a normal diet can significantly mitigate obesity and the lipotoxic microenvironment of mice. The sperm quality and fertility of the mice returned to normal levels, along with normal sperm acrosome integrity and structure in the testes; accordingly, there was no increased level of oxidative stress, abnormal spermatogenic cell apoptosis, and the expression of testosterone synthesis and Sertoli cell related proteins returned to normal. These results indicate that, at least to a certain extent, even if the specimen is obese during puberty, the testicular function and fertility of the mice could still be restored to normal levels after returning to a normal diet in adulthood.

In addition to the various mechanisms related to reduced fertility in our article, it is also possible that other mechanisms may lead the damaged fertility. It has demonstrated widely that decreased sirt1 causes mitochondrial dysfunction by increasing oxidative stress levels and apoptosis in male sperms, leading to infertility (55, 56). In addition, when it has demonstrated that seminal sirt1 expression was significantly lower in infertile men than fertile men, a recent study demonstrated that sirt1 can regulate acrosome biogenesis by modulating autophagic flux during spermiogenesis in mice (56, 57). Based on the results of MDA and apoptosis we tested, as well as related results of published studies, it is possible that sirt1 are an underlying mechanism for the association between high-fat diet and fertility.

In our present study, paternal obesity has been associated with low average litter weight. Previous study has demonstrated that paternal obesity induced by high-fat diet can affect offspring metabolic by means of epigenetic mechanisms (58). A recent work demonstrated paternal exposure to type 2 diabetes mediates intergenerational and transgenerational effects on the reproductive health of the offspring, especially on sperm quality (59). Accordingly, it is also possible that epigenetic mechanisms lead the inheritance of metabolic disturbances to the offspring. Further research will be carried out to reveal the underlying mechanism of damaged fertility in our future study.

In conclusion, this study confirms that obesity induced by a high-fat diet at different stages of sexual development is likely to cause disorders in glycolipid metabolism, disrupt the testicular structure, and damage the reproductive system. These above conclusions are verified by the increased number of spermatogenic cell apoptosis, impaired acrosome integrity, and the decreased expression of StAR and WT-1. A long-term high-fat diet resulted in more significant fertility damage. It is worth noting that the ectopic lipid accumulation, testicular structural and dysfunction, and impaired fertility caused by the high fat diet during puberty could be remarkably reversed by reverting to normal diet in adulthood. This finding strengthens the case for vigilance regarding adolescent obesity and supports the notion of intervention as soon as possible. Further research shall be carried out to reveal the underlying molecular mechanism of the recovery of fertility in mice following the reversion to a normal diet.

## Data Availability Statement

The original contributions presented in the study are included in the article/**Supplementary Material**. Further inquiries can be directed to the corresponding author.

## Ethics Statement

The animal study was reviewed and approved by the Animal Ethics Committee of Shandong Provincial Hospital.

## Author Contributions

XQ and MZ made the experiments and wrote the manuscript. MS, DL, and QG conducted research designed and performed the study. All authors contributed to the article and approved the submitted version.

## Funding

This work was supported by grants from the National Natural Science Foundation (81770860, 81471078 and 81641030) and the Key Research and Development Plan of Shandong Province (2017CXGC1214).

## Conflict of Interest

The authors declare that the research was conducted in the absence of any commercial or financial relationships that could be construed as a potential conflict of interest.

## Publisher’s Note

All claims expressed in this article are solely those of the authors and do not necessarily represent those of their affiliated organizations, or those of the publisher, the editors and the reviewers. Any product that may be evaluated in this article, or claim that may be made by its manufacturer, is not guaranteed or endorsed by the publisher.

## Supplementary Material

Supplementary Figure 1 The flowchart of animal experiment.
