# Supplementary figures and images for: Restoring Impaired Fertility Through Diet: Observations of Switching From High-Fat Diet During Puberty to Normal Diet in Adulthood Among Obese Male Mice
Source: Front Endocrinol (Lausanne). 2022 Apr 19;13:839034. doi: 10.3389/fendo.2022.839034 (PMC9063411; doi:10.3389/fendo.2022.839034)

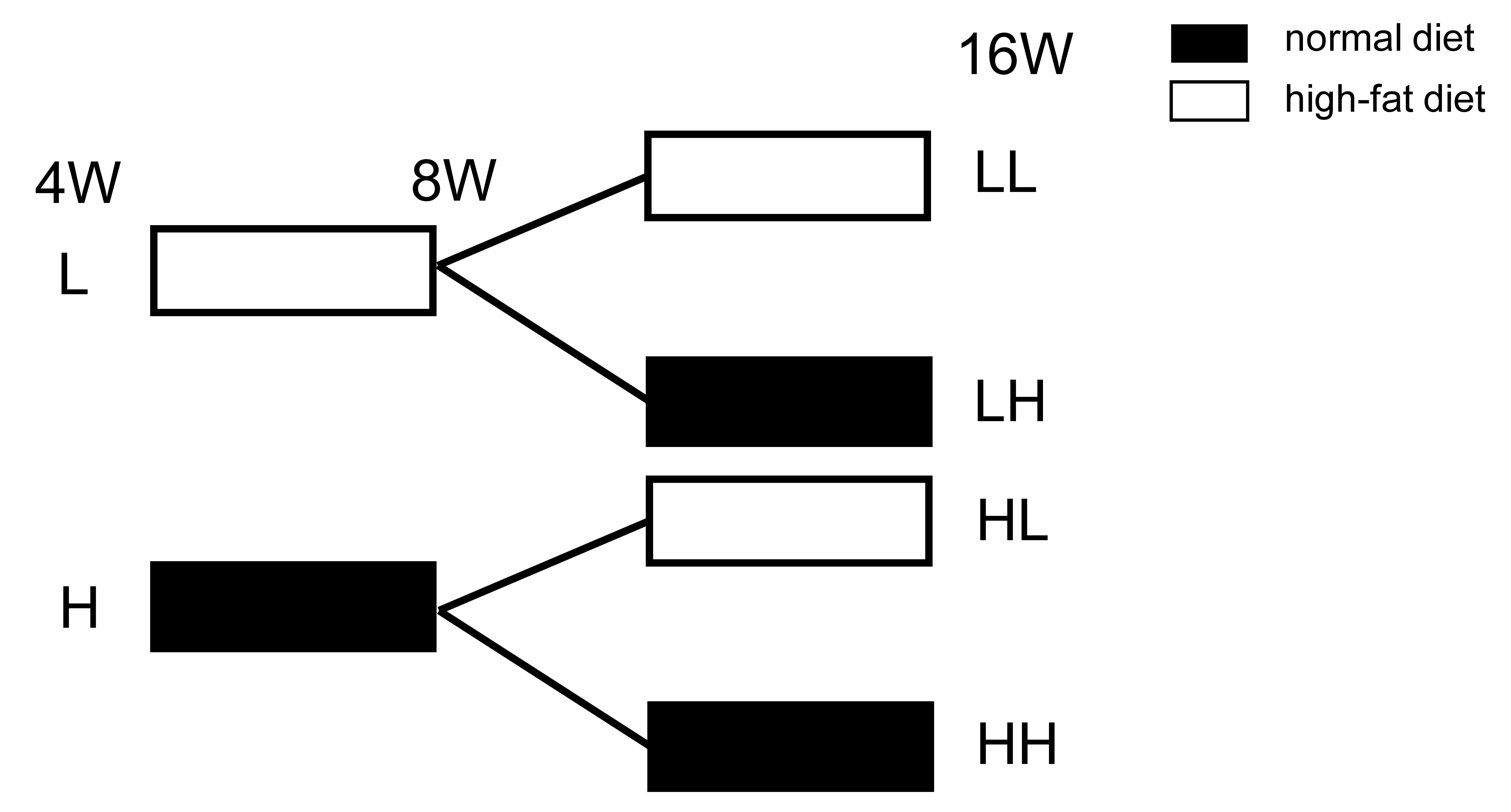

Supplement: Supplementary Figure 1 — The flowchart of animal experiment. [file Image_1.tif]
